# IFNγ Expression Correlates with Enhanced Cytotoxicity in CD8+ T Cells

**DOI:** 10.3390/ijms26147024

**Published:** 2025-07-21

**Authors:** Varsha Pattu, Elmar Krause, Hsin-Fang Chang, Jens Rettig, Xuemei Li

**Affiliations:** 1 Cellular Neurophysiology, Center for Integrative Physiology and Molecular Medicine (CIPMM), Saarland University, 66421 Homburg, Germany; varsha.pattu@gmail.com (V.P.); elmar.krause@uks.eu (E.K.); hsin-fang.chang@uks.eu (H.-F.C.); jrettig@uks.eu (J.R.); 2 Department of Neurology, NHC Key Laboratory of Diagnosis and Treatment on Brain Functional Diseases, The First Affiliated Hospital of Chongqing Medical University, No.1 Youyi Road, Yuzhong District, Chongqing 400016, China

**Keywords:** CD8+ T cells, interferon-gamma, CD107a, cytotoxicity, subcellular localization, CRTAM

## Abstract

CD8+ T lymphocytes (CTLs) act as serial killers of infected or malignant cells by releasing large amounts of interferon-gamma (IFNγ) and granzymes. Although IFNγ is a pleiotropic cytokine with diverse immunomodulatory functions, its precise spatiotemporal regulation and role in CTL-mediated cytotoxicity remain incompletely understood. Using wild-type and *granzyme B-mTFP* knock-in mice, we employed a combination of in vitro approaches, including T cell isolation and culture, plate-bound anti-CD3e stimulation, degranulation assays, flow cytometry, immunofluorescence, and structured illumination microscopy, to investigate IFNγ dynamics in CTLs. IFNγ expression in CTLs was rapid, transient, and strictly dependent on T cell receptor (TCR) activation. We identified two functionally distinct IFNγ-producing subsets: IFNγ^high^ (IFNγ^hi^) and IFNγ^low^ (IFNγ^lo^) CTLs. IFNγ^hi^ CTLs exhibited an effector/effector memory phenotype, significantly elevated CD107a surface expression (a marker of lytic granule exocytosis), and higher colocalization with cis-Golgi and granzyme B compared to IFNγ^lo^ CTLs. Furthermore, CRTAM, an early activation marker, correlated with IFNγ expression in naive CTLs. Our findings establish a link between elevated IFNγ production and enhanced CTL cytotoxicity, implicating CRTAM as a potential regulator of early CTL activation and IFNγ induction. These insights provide a foundation for optimizing T cell-based immunotherapies against infections and cancers.

## 1. Introduction

CD8+ cytotoxic T lymphocytes (CTLs) are critical immune sentinels that protect the host against infections and malignancies. Upon antigen recognition by specialized antigen-presenting cells (APCs), naive CD8+ T cells (T_N_s) undergo clonal expansion and differentiate into short-lived effector CTLs (T_E_s), which migrate to peripheral tissues and inflammatory sites [1,2]. Most T_E_s undergo apoptosis during the contraction phase [3], while a small subset persists as long-lived memory T cells (T_M_s). These memory populations consist of at least two distinct subsets: central memory (T_CM_) and effector memory (T_EM_) T cells [4,5]. In mice, short-lived T_E_s downregulate L-selectin (CD62L) but retain partial expression of IL-7 receptor alpha (CD127) [6], whereas long-lived T_M_s constitutively express CD127. T_CM_s exhibit high CD62L expression, while T_EM_s downregulate CD62L. Notably, under chronic infections or cancer, persistent antigen exposure can drive naive T cells into a dysfunctional state termed T cell exhaustion (T_EX_) [7]. Understanding the heterogeneity and function of CTL subsets is crucial to discern the complex dynamics of immune responses in various diseases [8].

CTLs eliminate target cells through lytic (granzymes and perforin) and non-lytic (IFNγ and TNF-α) mechanisms [9,10]. Their effector functions are regulated by transcription factors such as T-bet, Eomes, CRTAM (Class-I MHC-restricted T cell-associated molecule), T-box, and Runx family proteins [11,12,13]. The killing capacity of CTLs depends on cytotoxic granules (CGs) and degranulation efficiency [14]. CGs are secretory lysosomes (SGs) containing granzymes and perforin [15], enclosed by a lipid bilayer embedded with lysosome-associated membrane glycoproteins (LAMPs), including CD107a (LAMP-1), CD107b (LAMP-2), and CD63 (LAMP-3) [16]. Upon target recognition, CGs fuse with the plasma membrane, releasing their cytotoxic substances via exocytosis [13]. Since resting CTLs lack surface LAMPs, CD107a/b expression serves as a quantitative marker for degranulation [12]. Additionally, Fas ligand (FasL/CD95L), stored in specialized SGs, is externalized during degranulation to induce apoptosis in target cells [17]. Intriguingly, CTLs also express Fas receptor (Fas), enabling fratricidal killing (CTL fratricide)—a mechanism that eliminates effector T cells during immune contraction [17].

Beyond direct cytotoxicity, CTLs exert effector functions via interferon-gamma (IFNγ) and tumor necrosis factor-alpha (TNF-α) secretion. IFNγ is primarily produced by NK cells, Th1 cells, and CD8+ T cells [18,19]. In murine models of *Mycobacterium tuberculosis* infection, CD4+ and CD8+ T cells are the dominant IFNγ producers in vivo [20]. IFNγ is a pleiotropic cytokine with dual roles—exhibiting antiviral [21,22] and antitumor activity while paradoxically promoting tumor progression in certain contexts [23,24,25]. Mechanistically, IFNγ signaling upregulates interferon regulatory factor-1 (IRF-1), which enhances MHC-I/II expression, rendering target cells more susceptible to CTL attack [26]. As a proinflammatory cytokine, IFNγ also activates macrophages to secrete TNF-α and IL-6 [27,28,29].

In this study, we investigated IFNγ production, subcellular localization, cellular subtypes, and cytotoxic function in IFNγ-expressing CTLs. We found that naive CD8+ T cells rapidly acquire IFNγ production within 2 h of TCR activation, whereas GzmB expression is restricted to effector CTLs with mature immune synapses [30]. Activated CTLs exhibited transient, TCR-dependent IFNγ expression, contrasting with pre-stored GzmB in resting CTLs. Strikingly, IFNγ+ CTLs segregated into IFNγ^hi^ and IFNγ^lo^ subsets, while GzmB^+^ CTLs were homogeneous. IFNγ^hi^ cells displayed a T_E_/T_EM_ phenotype (low CD62L), whereas IFNγ^lo^ cells resembled T_CM_ (high CD62L). Degranulation assays revealed that IFNγ^hi^ CTLs exhibited significantly higher surface CD107a than IFNγ^lo^ cells, suggesting superior lytic activity. Furthermore, IFNγ^hi^ CTLs showed pronounced colocalization with cis-Golgi and GzmB+ vesicles, reinforcing the link between IFNγ expression and cytotoxicity. Finally, comparative analysis of IFNγ and CRTAM expression in naive vs. activated CD8+ T cells suggested that CRTAM may regulate early T cell activation and IFNγ production.

## 2. Results

### 2.1. Transient IFNγ Expression Depends on TCR Reactivation in Resting CTLs

To determine whether interferon-gamma (IFNγ) expression is constitutive or regulated in activated CTLs, we analyzed intracellular IFNγ dynamics following TCR re-stimulation as previously reported [31]. Days 3–5 activated CTLs, with or without re-stimulation, were fixed, permeabilized (BD Cytofix/Cytoperm™, San Jose, CA, USA), and stained for IFNγ-Alexa488 and GzmB-Alexa647 (without Golgi transport inhibition). Flow cytometry data were acquired and analyzed using FlowJo v10.

**Figure 1:**
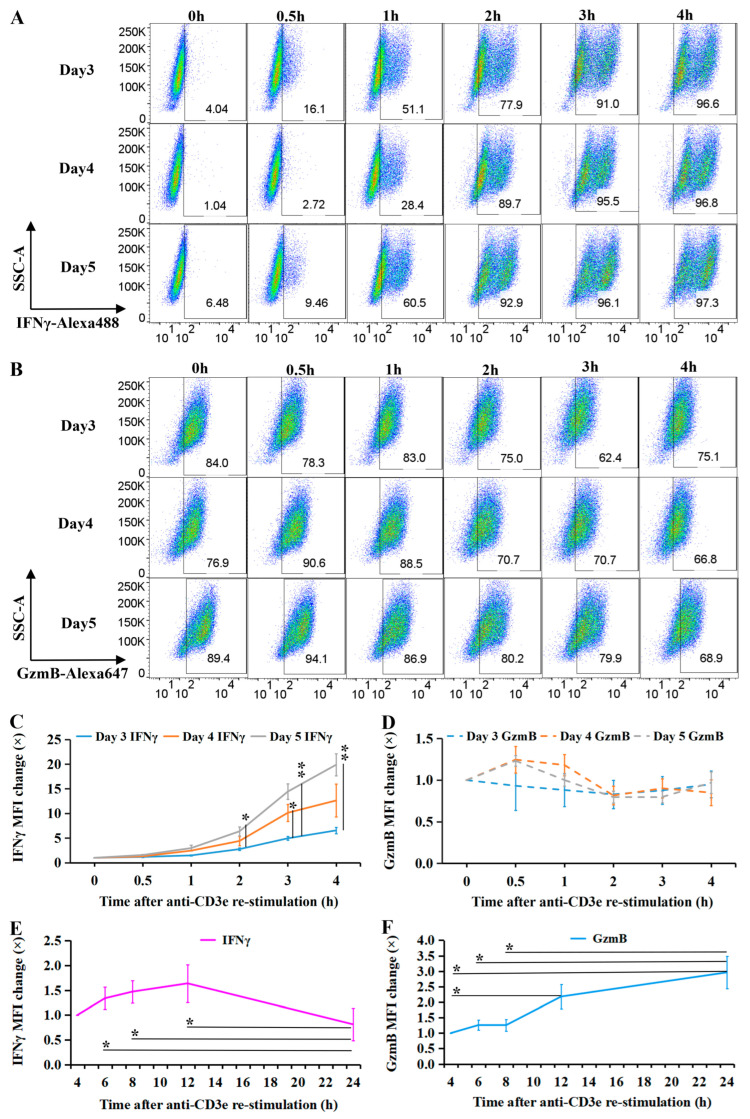
Intracellular IFNγ and granzyme B (GzmB) expression in activated CTLs. (**A**–**D**) WT CTLs (days 3–5 post-activation) were re-stimulated with plate-bound anti-CD3e antibody (10 μg/mL) and analyzed for intracellular IFNγ and GzmB expression over 0–4 h. (**A**,**B**) Pseudocolor plots depict fluorescence intensity of rat anti-mouse IFNγ-Alexa488 (*x*-axis) and GzmB-Alexa647 (*x*-axis) against side scatter area (SSC-A, *y*-axis). (**C**,**D**) Median fluorescence intensity (MFI) of IFNγ-Alexa488 and GzmB-Alexa647. (**E**,**F**) Temporal changes in IFNγ and GzmB expression in re-stimulated CTLs. MFI dynamics of IFNγ-Alexa488 and GzmB-Alexa647. Data are mean ± SEM (*N* ≥ 3, number of experimental repeats). Statistical significance was determined by one-way ANOVA and unpaired *t*-test (* *p* < 0.05, ** *p* < 0.01).

IFNγ- and GzmB-expressing CTLs were shown in pseudocolor plots in 4 h TCR re-stimulation (Figure 1A,B). In unstimulated CTLs, IFNγ was nearly undetectable, but its expression increased rapidly upon TCR re-stimulation (Figure 1A). A large amount of GzmB existed before re-stimulation and had less variation after TCR re-stimulation (Figure 1B). The expression level of IFNγ and GzmB in CTLs exhibited a maturation-dependent pattern and distinct kinetics. IFNγ expression (measured by median fluorescence intensity, MFI) showed a transient peak (Figure 1C), with day 5 CTLs displaying faster upregulation than day 3 CTLs at 2 h (*p* = 0.020), 3 h (*p* = 0.004), and 4 h (*p* = 0.004). Day 4 CTLs showed IFNγ MFI kinetics comparable to day 5 CTLs but significantly faster than day 3 at 3 h (*p* = 0.044). In contrast, GzmB MFI remained stable across days 3–5 CTLs during the 4-h re-stimulation period (Figure 1D).

Longitudinal analysis revealed divergent temporal patterns: IFNγ levels went up in 12 h, then back down in the next 12 h, while GzmB accumulated continuously. IFNγ MFI was significantly elevated at 6 h, 8 h, and 12 h compared to 24 h (*p* = 0.021, *p* = 0.017, and *p* = 0.021, respectively) (Figure 1E). Conversely, GzmB MFI showed progressive accumulation, with significantly higher levels at 24 h versus 4 h, 6 h, and 8 h (*p* = 0.021, *p* = 0.037, and *p* = 0.039), and at 12 h versus 4 h (*p* = 0.042) (Figure 1F).

In conclusion, in activated CTLs, the expression of IFNγ and GzmB exhibited a maturation-dependent pattern and distinct kinetics. IFNγ expression is transient, but GzmB expression lasts longer compared to IFNγ after TCR (re)activation.

### 2.2. CTL Subtype Dynamics upon TCR Re-Stimulation

Cytotoxic T lymphocytes (CTLs) comprise distinct functional subsets, including effector cells (T_E_s), effector memory cells (T_EM_s), and central memory cells (T_CM_s). To determine whether IFNγ expression is subtype-specific and whether TCR re-stimulation alters the subtype composition of cultured CTLs, we stimulated activated CTLs with 10 μg/mL plate-bound anti-CD3e [31] for 0.5–4 h and analyzed subtype markers (CD44^hi^ for T_E_s/T_EM_s, CD62^hi^ for T_CM_s) by flow cytometry at multiple timepoints.

**Figure 2:**
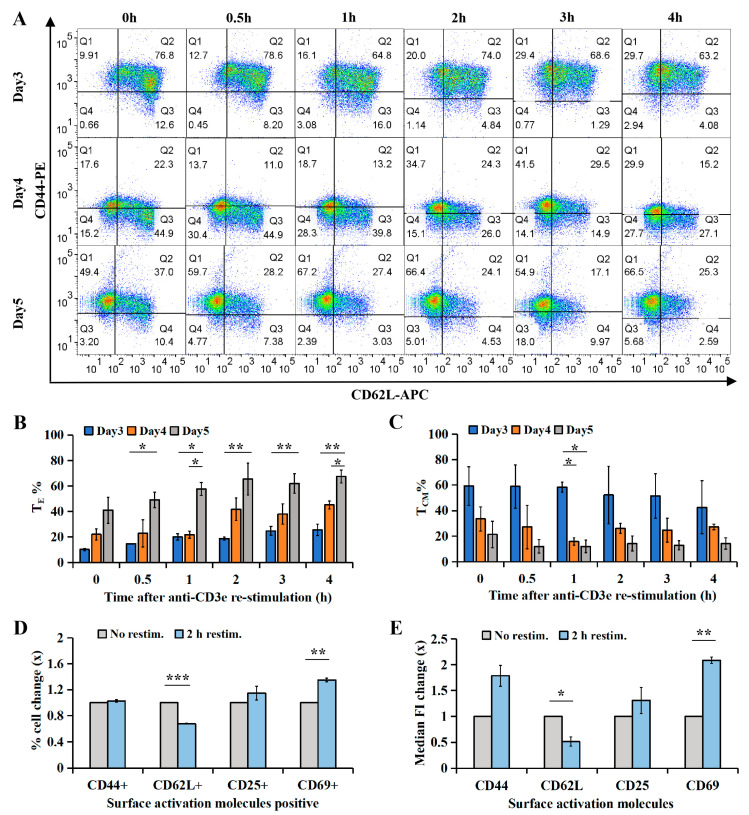
Surface activation marker expression profiles in CTLs analyzed by flow cytometry. (**A**–**C**) WT CTLs (days 3–5 post-activation) were re-stimulated with plate-bound anti-CD3e antibody (10 μg/mL) for 0–4 h and stained for CD44 and CD62L. (**A**) Pseudocolor plots depict fluorescence intensity of rat anti-mouse CD62L-APC (*x*-axis) and CD44-PE (*y*-axis). (**B**,**C**) Frequencies of T_E_ and T_CM_ populations. (**D**,**E**) WT CTLs (day 5 post-activation) were re-stimulated with plate-bound anti-CD3e (10 μg/mL) for 2 h and analyzed for multiple activation markers. (**D**) Fold change in CD44+, CD62L+, CD25+, and CD69+ cell populations. (**E**) MFI change of CD44-PE, CD62L-APC, CD25-PE, and CD69-APC before and after 2 h re-stimulation. Data represent mean ± SEM (*N* ≥ 3, number of experimental repeats). Statistical analysis was performed using one-way ANOVA and unpaired *t*-test (* *p* < 0.05, ** *p* < 0.01, *** *p* < 0.001).

Neither the percentage of CD44+ cells (Supplementary Materials Figure S1A) nor CD44 MFI (Supplementary Materials Figure S1B) changed significantly during 4 h of re-stimulation, regardless of culture day (days 3–5), indicating that the overall effector differentiation status of the culture remained stable, independent of TCR re-stimulation or culture duration. In contrast, the CD62L+ cell percentage (Supplementary Materials Figure S1C) and CD62L MFI (Supplementary Materials Figure S1D) declined quickly upon re-stimulation (*p* < 0.05), indicating a transition from T_CM_s to T_EM_s (Figure 2A–C).

Pseudocolor plots in Figure 2A revealed subtype distribution. Cells in Q1 (CD44+CD62-) were T_E_s_/EM_s, those in Q2 (CD44+CD62+) were T_CM_s, and those in Q3 (CD44-CD62+) were T_N_s. Day 5 CTLs exhibited significantly more T_E_s/T_EM_s than day 3 CTLs at 4 h and day 4 CTLs at 1 h and 4 h (*p* < 0.05; Figure 2B). Concurrently, the T_CM_ percentage was lower in day 5 compared to day 3 and day 4 CTLs at 1 h (*p* < 0.05; Figure 2C), confirming that longer culture promotes T_E/EM_ differentiation upon re-stimulation.

Screening of activation markers (CD25, CD44, CD62L, CD69) in day 5 CTLs revealed robustly decreased CD62L and increased CD69 in both cell percentage and MFI (Figure 2D,E) after 2 h re-stimulation, while no significant changes were found in CD44 or CD25, indicating that CD62L downregulation and CD69 upregulation are robust indicators of TCR-driven T_CM_-to-T_EM_ transition.

### 2.3. Cellular Subtype and Cytotoxicity Analysis of IFNγ^hi^ and IFNγ^lo^ CTLs

**Figure 3:**
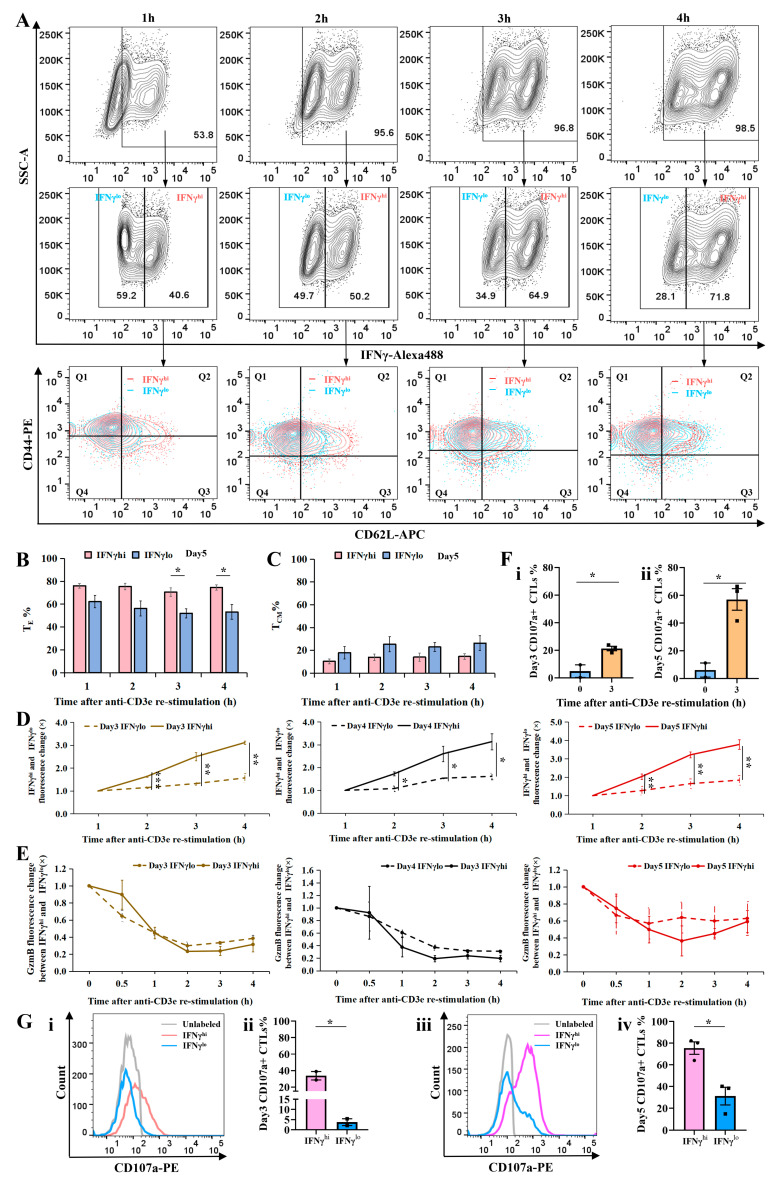
Characterization of IFNγ^hi^ and IFNγ^lo^ CTL subsets and their cytotoxic potential. WT CTLs were re-stimulated with plate-bound anti-CD3e antibody for 0–4 h and processed for flow cytometry analysis. (**A**–**C**) Restimulated CTLs were surface-stained with anti-CD44-APC and anti-CD62L-PE, followed by intracellular staining for IFNγ-Alexa488. (**D**,**E**) Restimulated CTLs were intracellularly stained for IFNγ-Alexa488 and GzmB-Alexa647. (**F**,**G**) CTLs were surface-stained with anti-CD107a-PE during 3 h restimulation, followed by intracellular IFNγ staining. (**A**) Gating strategy and surface marker expression profiles of IFNγ^hi^ vs. IFNγ^lo^ CTLs. (**B**) Percentage of effector T cells (T_E_s) in each subset. (**C**) Percentage of central memory T cells (T_CM_s) in each subset. (**D**) IFNγ fluorescence intensity in IFNγ^hi^ vs. IFNγ^lo^ subsets. (**E**) GzmB fluorescence intensity in IFNγ^hi^ vs. IFNγ^lo^ subsets. (**F**,**G**) Degranulation capacity assessed by CD107a surface expression. (F) Percentage of CD107a+ CTLs pre- and post-stimulation. (**G**) (**i**,**iii**): CD107a expression in IFNγ^hi^ vs. IFNγ^lo^ CTLs at days 3 and 5. (**ii**,**iv**): CD107a fluorescence intensity in IFNγ^hi^ vs. IFNγ^lo^ CTLs at days 3 and 5. Data represent mean ± SEM (*N* ≥ 2 independent experiments). Statistical significance was determined by unpaired *t*-test (* *p* < 0.05, ** *p* < 0.01, *** *p* < 0.001).

To determine whether IFNγ expression correlates with cytotoxic T lymphocyte (CTL) effector function, we analyzed reactivated CTLs, which exhibited distinct clusters of high (IFNγ^hi^) and low (IFNγ^lo^) IFNγ expression (Figure 1A and Figure 3A, row 1). Using a fluorescence intensity threshold of 10^3^, we classified IFNγ-positive CTLs into IFNγ^hi^ and IFNγ^lo^ subsets (Figure 3A, row 2). Longer re-stimulation led to increased effector T cell (T_E_) generation (Figure 2B and Figure 3A row3). The IFNγ^hi^ subset generated a significantly higher ratio of T_E_s than the IFNγ^lo^ subset at 3 and 4 h of re-stimulation (Figure 3B).

Although IFNγ^hi^ and IFNγ^lo^ CTLs showed comparable CD44 expression, IFNγ^hi^ CTLs exhibited significantly lower CD62L levels compared to IFNγ^lo^ CTLs (Supplementary Materials Figure S2). The proportion of T_CM_s was slightly higher in IFNγ^lo^ CTLs, though not statistically significant (Figure 3C). When assessing IFNγ production kinetics, IFNγ^hi^ CTLs displayed markedly higher IFNγ MFI than IFNγ^lo^ CTLs at 2, 3, and 4 h post-stimulation (Figure 3D), whereas GzmB MFI did not differ significantly between the two subsets (Figure 3E).

Extended TCR stimulation revealed a pronounced divergence in IFNγ fluorescence between IFNγ^hi^ and IFNγ^lo^ CTLs by 24 h (*p* = 0.001), while GzmB fluorescence remained comparable (Supplementary Materials Figure S3). In IFNγ^hi^ CTLs, IFNγ fluorescence peaked at 12 h before declining significantly by 24 h (vs. 4 h, *p* = 0.042 and *p* = 0.0025). In contrast, IFNγ^lo^ CTLs exhibited a gradual increase, peaking at 12 h without subsequent decline, and showed higher fluorescence at 6, 12, and 24 h than at 4 h (*p* = 0.0483, *p* = 0.0112, and *p* = 0.0063, Supplementary Materials Figure S3A). GzmB fluorescence in IFNγ^hi^ CTLs rose significantly at 6, 8, and 24 h (vs. 4 h, *p* = 0.0022, *p* = 0.0075, and *p* = 0.0213), whereas in IFNγ^lo^, CTLs showed increased GzmB only at 12 and 24 h (vs. 4 h, *p* = 0.0246 and *p* = 0.0171, Supplementary Materials Figure S3B).

To evaluate cytotoxic capacity, we measured CD107a surface expression, a marker of degranulation. Unstimulated CTLs exhibited minimal CD107a expression (~5%), but upon 3-h re-stimulation, levels surged to 21.33% (day 3) and 57% (day 5), with day 5 showing significantly higher degranulation than day 3 (*p* = 0.0109; Figure 3F), suggesting that degranulation capacity escalates with CTL maturation. Notably, IFNγ^hi^ CTLs consistently displayed higher CD107a expression than IFNγ^lo^ CTLs (Figure 3G). Flow cytometry histograms depicted CD107a fluorescence intensity in unlabeled (gray), IFNγ^hi^ (pink), and IFNγ^lo^ (blue) CTLs (Figure 3G(i),(iii)). On day 3, 33.75% of IFNγ^hi^ CTLs underwent degranulation versus only 3.76% of IFNγ^lo^ CTLs (Figure 3G(ii)). By day 5, degranulation frequencies rose to 75.47% (IFNγ^hi^) and 31.13% (IFNγ^lo^) (Figure 3G(iv)). These results suggests that cytotoxic activity correlates positively with IFNγ expression and cellular maturation, underscoring the functional superiority of IFNγ^hi^ CTLs.

### 2.4. Subcellular Localization Analysis of IFNγ in IFNγ^hi^ and IFNγ^lo^ CTLs

To characterize the spatial distribution of IFNγ in cytotoxic T lymphocytes (CTLs), we analyzed the Pearson’s correlation coefficient (PCC) between endogenous IFNγ, cis-Golgi, and granzyme B (GzmB) in IFNγ^hi^ and IFNγ^lo^ subsets.

**Figure 4:**
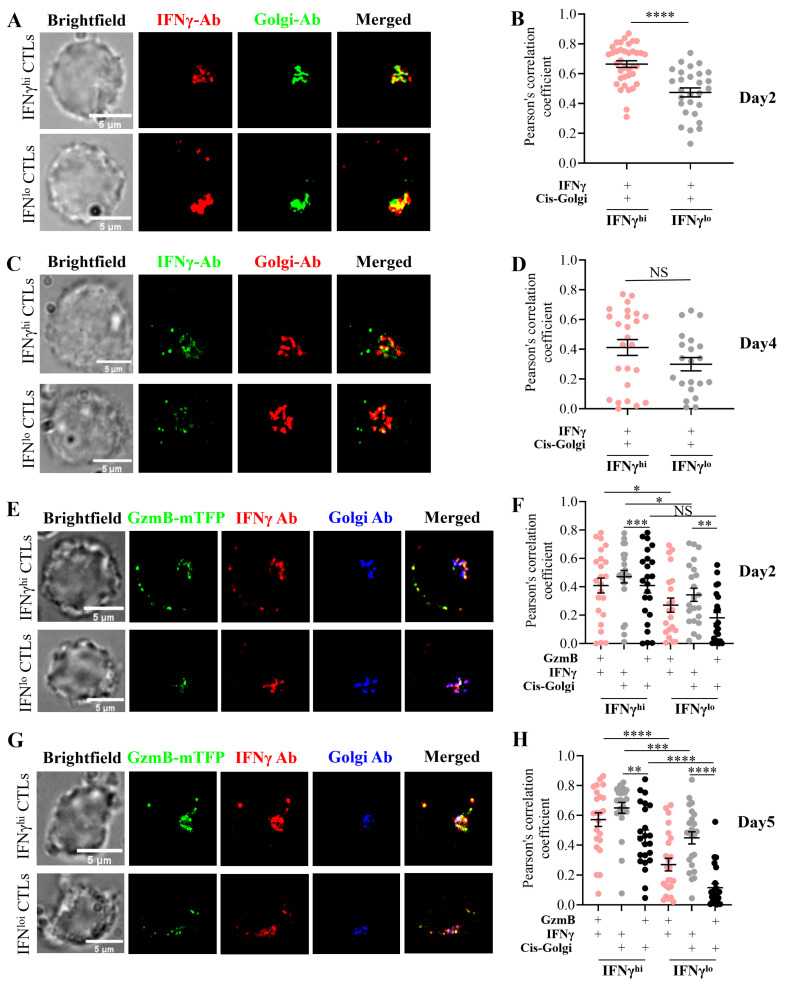
Subcellular localization of IFNγ in IFNγ^hi^ and IFNγ^lo^ CTL subsets. (**A**–**H**) WT or *GzmB-mTFP* KI CTLs were re-stimulated with plate-bound anti-CD3e (10 μg/mL) (for day 2: 4 h re-stimulation, day 4 and day 5: 2 h re-stimulation) and processed for super-resolution microscopy (SIM). Imaging and analysis: Cells were stained for IFNγ, GzmB, and cis-Golgi marker (GM130); IFNγ^hi^ vs. IFNγ^lo^ subsets were imaged using optimized laser power and EMCCD gain settings. Colocalization analysis was performed using Fiji-win64 software. Quantitative comparisons and graphing were performed using GraphPad Prism 10.2.3. Experimental details: (**A**,**B**) day 2 WT CTLs: Primary antibodies: Rat anti-mouse IFNγ (1:200), mouse anti-GM130 (1:100). Secondary antibodies: Alexa647 chicken anti-rat IgG (1:800), Alexa568 goat anti-mouse IgG (1:400). Imaging parameters: IFNγ^lo^: 561 nm laser (1.5%, gain 80); 647 nm laser (1.5%, gain 50). IFNγ^hi^: 561 nm laser (1.2%, gain 80); 647 nm laser (0.6%, gain 25). (**B**) Pearson’s correlation coefficient analysis. (**C**,**D**) Day 4 WT CTLs: Primary antibodies: Rat anti-mouse IFNγ-Alexa488 (1:200), mouse anti-GM130 (1:100). Secondary antibody: Goat anti-mouse-Alexa647 (1:1000). (**D**) Pearson’s correlation coefficient for IFNγ and cis-Golgi colocalization. (**E**–**H**) *GzmB-mTFP* KI CTLs: Primary antibodies: Rat anti-mouse IFNγ (1:200), mouse anti-GM130 (1:100). Secondary antibodies: Alexa647 chicken anti-rat IgG (1:800), Alexa568 goat anti-mouse IgG (1:400). (**E**,**F**) Day 2 CTL imaging parameters: IFNγ^hi^: 488 nm (22%, gain 80); 568 nm (1.2%, gain 80); 647 nm (0.7%, gain 40). IFNγ^lo^: 488 nm (26%, gain 80); 568 nm (1.2%, gain 80); 647 nm (1.5%, gain 80). (**G**,**H**) Day 5 CTL imaging parameters: IFNγ^hi^: 488 nm (18%, gain 80); 561 nm (1.2%, gain 80); 647 nm (0.3%, gain 20). IFNγ^lo^: 488 nm (16%, gain 80); 561 nm (1.5%, gain 80); 647 nm (1.0%, gain 80). (**F**,**H**) Pearson’s correlation coefficient analyses. Statistical analysis: Data represent mean ± SEM. Significance was determined by one-way ANOVA with *t*-tests (* *p* < 0.05, ** *p* < 0.01, *** *p* < 0.001, **** *p* < 0.0001; NS, not significant).

Wild-type (WT) CTLs were re-stimulated with plate-bound aCD3e and stained for endogenous IFNγ and cis-Golgi. Super-resolution structured illumination microscopy (SIM) was performed using Zen software, and PCC was quantified with Fiji. IFNγ exhibited extensive colocalization with cis-Golgi in WT CTLs. Significantly higher colocalization was displayed in IFNγ^hi^ CTLs compared to IFNγ^lo^ CTLs after 4 h of re-stimulation on day 2 (Figure 4A,B), while no significant difference was observed in the PCC of IFNγ and Golgi between IFNγ^hi^ and IFNγ^lo^ CTLs at 2 h post-stimulation on day 4 (Figure 4C,D).

To determine whether IFNγ traffics to CGs, we analyzed IFNγ and GzmB colocalization in *GzmB-mTFP* knock-in (KI) CTLs on days 2 and 5 post-stimulation. GzmB is a marker for CGs. SIM imaging (Figure 4E,F) and Fiji-based quantification (Figure 4G,H) revealed that a subset of IFNγ localized in GzmB+ CGs, with IFNγ^hi^ CTLs exhibiting significantly greater IFNγ-GzmB association than IFNγ^lo^ CTLs (Figure 4F,H).

IFNγ showed robust cis-Golgi localization in *GzmB-mTFP* KI CTLs, with IFNγ^hi^ CTLs displaying higher enrichment than IFNγ^lo^ CTLs (Figure 4F,H). In contrast, GzmB exhibited minimal Golgi association, though IFNγ^hi^ CTLs on day 5 demonstrated a slightly higher PCC of GzmB and Golgi than IFNγ^lo^ CTLs (Figure 4H). No such difference was observed on day 2 (Figure 4F).

These findings demonstrate that the IFNγ^hi^ subset retains higher IFNγ expression and greater IFNγ localization within CGs compared to IFNγ^lo^ CTLs. Newly synthesized IFNγ accumulates more prominently in the Golgi apparatus than GzmB following acute TCR stimulation. The preferential trafficking of IFNγ to CGs in IFNγ^hi^ CTLs suggests a direct role in cytotoxicity, consistent with their enhanced effector function. IFNγ expression is dynamically regulated, with transient Golgi-associated production preceding effector granule loading. Together, these data support a model wherein IFNγ^hi^ CTLs achieve superior cytotoxic activity through coordinated IFNγ synthesis, Golgi processing, and granule loading.

### 2.5. Temporal Regulation of CRTAM and IFNγ in Naive and Activated CTLs

To investigate the relationship between activation markers and IFNγ expression during early and late T cell activation, we analyzed IFNγ, CRTAM, CD62L, CD69, and CD25 expression in wild-type CD8+ T cells at day 0 (naive) and day 5 (activated) following TCR stimulation or re-stimulation.

**Figure 5:**
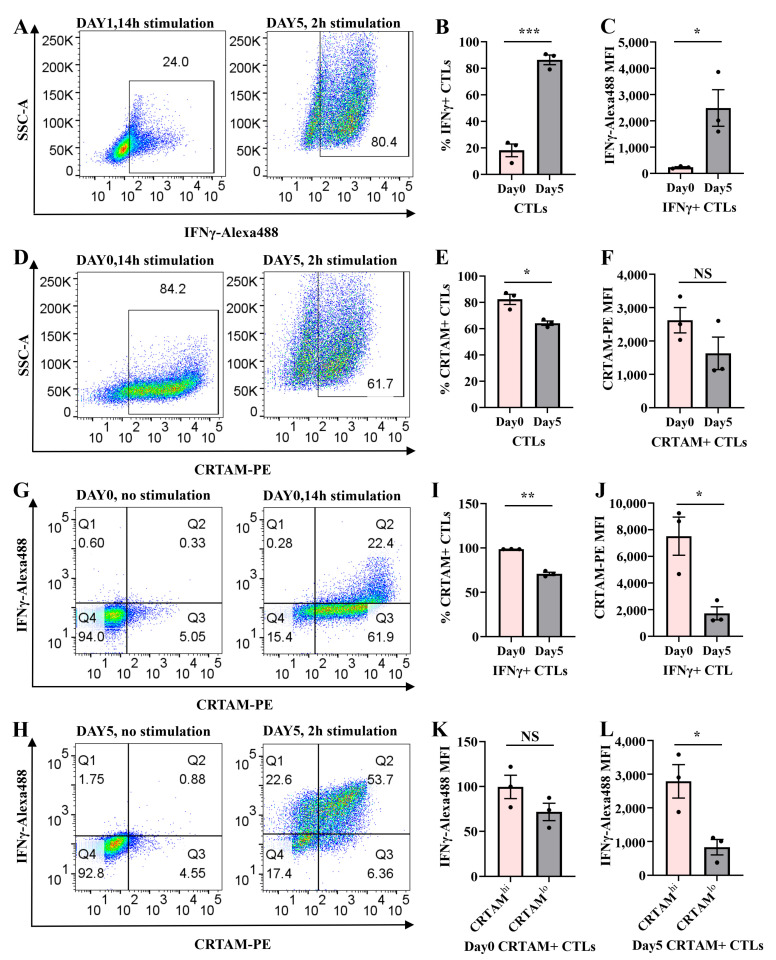
Co-expression analysis of CRTAM and IFNγ in activated CTLs by flow cytometry. Experimental design: Day 0 CTLs: Stimulated with plate-bound anti-CD3e (10 μg/mL) and anti-CD28 (5 μg/mL) for 14 h. Day 5 CTLs: Re-stimulated with plate-bound anti-CD3e (10 μg/mL) for 2 h in 96-well plates. Staining: Anti-CRTAM-PE and anti-IFNγ-Alexa488 antibodies. Flow cytometry analysis: (**A**) Representative pseudocolor plots of IFNγ expression in day 0 vs. day 5 CTLs. (**B**) Percentage of IFNγ+ CTLs. (**C**) MFI of IFNγ-Alexa488. (**D**) Representative pseudocolor plots of CRTAM expression. (**E**) Percentage of CRTAM+ CTL populations. (**F**) MFI of CRTAM-PE. Co-expression analysis: (**G**,**H**) Quadrant plots showing IFNγ and CRTAM co-expression patterns. (**I**) Percentage of CRTAM+ CTLs in IFNγ+ CTLs. (**J**) MFI of CRTAM-PE in IFNγ+ CTLs. (**K**,**L**) MFI of IFNγ-Alexa488 in CRTAM^hi^ vs. CRTAM^lo^ subsets. Statistical analysis: Data represent mean ± SEM (N = 3 independent experiments). Significance was determined by unpaired *t*-test (* *p* < 0.05, ** *p* < 0.01, *** *p* < 0.001; NS, not significant).

Both CRTAM and IFNγ were upregulated upon TCR stimulation (Figure 5A–F, Supplementary Materials Figure S4A–C). CRTAM+ CTLs increased from 5.93% (1 h) to a peak of 96.6% (24 h) before declining to 10.4% by 72 h, while IFNγ+ CTLs rose from 5.24% (1 h) to 35.6% (24 h) and subsequently decreased to 9.62% (72 h) (Supplementary Materials Figure S4B). MFI for both molecules peaked at 24 h and became nearly undetectable by 72 h (Supplementary Materials Figure S4C). Notably, day 2 and day 3 CTLs regained CRTAM and IFNγ expression upon re-stimulation (4 h and 3 h, respectively), confirming their transient, activation-dependent regulation (Supplementary Materials Figure S4A).

Day 5 CTLs exhibited significantly higher percentages of IFNγ+ CTLs and IFNγ MFI than day 0 CTLs (Figure 5A–C), whereas naive CTLs had a greater proportion of CRTAM+ CTLs (Figure 5D,E). CRTAM MFI, however, did not differ significantly between day 0 and day 5 (Figure 5F). Quadrant analysis of pseudocolor plots revealed distinct co-expression patterns.

In day 0 CTLs, only 20–25% of CRTAM+ CTLs expressed IFNγ, but nearly 100% of IFNγ+ CTLs were CRTAM+ after 14 h stimulation (Figure 5G,I; Supplementary Materials Figure S4A). In day 5 CTLs, ~71% of IFNγ+ CTLs co-expressed CRTAM (Figure 5H,I).

Naive IFNγ+ CTLs also displayed higher CRTAM MFI than their day 5 counterparts (Figure 5J). Strikingly, CRTAM^hi^ CTLs produced more IFNγ than CRTAM^lo^ subsets (Figure 5K,L), underscoring a positive correlation between CRTAM and IFNγ.

CRTAM upregulation in naive CTLs occurred as rapidly as CD69 induction, preceding CD25 expression (1–6 h; Supplementary Materials Figure S4A,D,G). Conversely, CD62L levels declined post-stimulation (Supplementary Materials Figure S4J). By day 2, CTLs entered contraction/memory phases, marked by rising CD62L and declining CD69/CD25 (Supplementary Materials Figure S4E,F,I,L). With TCR re-stimulation, day2/3 CTLs were marked with reducing CD62L and increasing CD69/CD25 (Supplementary Materials Figure S4E,F,H,I,L), while the percentage of CD25 did not change (Supplementary Materials Figure S4K).

Our findings demonstrate that CRTAM and IFNγ are transiently expressed in a TCR-dependent manner. CRTAM serves as an early activation marker and correlates with IFNγ production in naive CTLs. CRTAM, CD62L, CD69, and CD25 collectively define CTLs’ activation states. These results position CRTAM as a key early responder during primary CTLs’ activation, functionally linked to IFNγ-mediated effector responses.

## 3. Discussion

Effector CD8+ T cells (CTLs) are central to antimicrobial and antitumor immunity, employing both lytic and non-lytic cytotoxic mechanisms. While their lytic function is mediated by GzmB and perforin is well-characterized [30,32], the role of interferon-gamma (IFNγ) in CTL-mediated cytotoxicity remains less defined. Our study elucidates the dynamic expression profile of IFNγ in activated CTLs and its functional contribution to their cytotoxic potential.

We demonstrate that IFNγ expression in CTLs is rapidly induced upon TCR activation, peaking early before declining transiently compared to the sustained expression of GzmB (Figure 1A–F). This transient expression coincided with CTL maturation, as evidenced by the progressive shift toward effector T cell (T_E_) phenotypes and the downregulation of CD62L (Figure 2). Notably, CTLs segregated into distinct IFNγ^hi^ and IFNγ^lo^ subsets, with the former exhibiting a stronger effector phenotype (Figure 3A–C).

Intriguingly, IFNγ^hi^ CTLs displayed significantly enhanced degranulation capacity (Figure 3D,G) despite comparable GzmB levels to IFNγ^lo^ CTLs (Figure 3E), suggesting that IFNγ potentiates lytic cytotoxicity independently of intracellular GzmB expression level. This was further supported by super-resolution imaging, which revealed greater colocalization of IFNγ with GzmB-positive cytotoxic granules in IFNγ^hi^ CTLs (Figure 4E–H). These findings position IFNγ not only as an immunomodulatory cytokine but also as a direct contributor to the lytic efficiency of CTLs. This finding is aligned with our previous research demonstrating that lytic IFNγ is stored within GzmB-containing cytotoxic granules and co-secreted at the immune synapse by effector CD8+ T cells, a mechanism that enhances cytotoxic T lymphocytes’ (CTLs) killing capacity [33]. Bhat et al. (2017) demonstrated that autocrine IFNγ production by CTLs enhances their motility and facilitates the killing of primary target keratinocytes, both in vitro and in vivo. This finding highlights the critical dependence of CD8+ T cell cytotoxic function on local IFNγ signaling, which has important implications for immunotherapy targeting chronic viral infections and cancers [34].

The mechanisms governing IFNγ production in CTLs remain incompletely understood. Our data highlight CRTAM as a key early activation marker that correlates with IFNγ expression. CRTAM was upregulated transiently in naive CTLs upon TCR stimulation, preceding IFNγ induction (Figure 5; Supplementary Materials Figure S3). Nearly all IFNγ+ CTLs co-expressed CRTAM during primary activation (Supplementary Materials Figure S3A), and CRTAM^hi^ subsets produced significantly more IFNγ than CRTAM^lo^ cells (Figure 5K,L).

The role of CRTAM in CTL biology aligns with its known functions in promoting IFNγ production in CD8+ T cells and CD4+ T cells [35,36], in regulating CD4+ T cell polarization, and in determining the CD4+ cytotoxic T lymphocyte lineage [35,37]. Its interaction with CADM1 may facilitate early CTL priming by retaining activated cells in lymphoid tissues [11], while its decline in day 5 CTLs (Figure 5I,J) suggests a stage-specific role in bridging early activation with effector differentiation.

While our work establishes a link between IFNγ expression and CTL cytotoxicity, further studies are needed to clarify whether IFNγ enhances lytic function through blocking IFNγ release from CTLs and in vivo autoimmune or cancer animal models are needed to verify their functional difference. Meanwhile, IFNγ is transported in and secreted from cytotoxic granules (CGs), at the immune synapse, which could increase the target cell sensitization to CTLs’ killing. This finding is helpful to explain the relation of IFNγ and CD107a (a marker on CGs), or the mechanism of IFNγ expression contributes to CTLs’ cytotoxicity. The results are proven by a combination of in vivo and in vitro experiments and are going to be published in the near future. Finally, the origin (mechanism) and physiological role of this bimodal expression should be further addressed in future studies. The molecular pathways connecting CRTAM to IFNγ regulation warrant further investigation. Single-cell RNA sequencing could delineate the transcriptional programs distinguishing IFNγ^hi^ and IFNγ^lo^ subsets [38,39,40], offering insights into their functional specialization. The benefit of this bimodal expression might be that the IFNγ^hi^ (effector-like) CTLs are sent to kill target cells efficiently, and IFNγ^lo^ (memory-like) CTLs are the backup, which keep proliferating and replenishing the effector CTL pool and recruit more immune cells to support effector CTLs in the first line. This is a very good strategy to kill pathogens without sacrificing all of the CTLs simultaneously and to continue gaining energy and support for the later fight to guarantee final success in removing all antigens.

## 4. Methods

### 4.1. Mice

Wild-type (WT) mice were purchased from Charles River, and *granzyme B-mTFP* knock-in (*GzmB-mTFP* KI) mice were generated as previously described [30]. All experimental procedures were conducted in compliance with regulations of the state of Saarland (Landesamt für Verbraucherschutz (Halle, Germany), AZ.: 2.4.1.1 and 11/2021).

### 4.2. Cell Isolation

Mice were anesthetized with CO_2_ and executed by cervical dislocation. The left abdominal cavity was exposed, and the spleen was carefully removed, placed on a 70 μm cell strainer (Corning Life Sciences, Singapore), and ground. The grinding slurry remaining on the strainer was rinsed with RPMI medium, and the cell suspension (10 mL) was collected into a 15 mL sterile centrifuge tube (Corning Life Sciences) and centrifuged (6 min, 1100 rpm without a break) to wash the splenocytes. Washed splenocytes were mixed and incubated with 1 mL erythrocyte lysis buffer (components: H_2_O, 100 mL, NH_4_Cl, 0.829 g, (155 mM), KHCO_3_, 0.1 g, (10 mM), EDTA from 50 mM stock-260 μL, pH 7.4 (0.1 mM)) for 30 s to lyse the erythrocytes therein (Bzeih, 2016) [41]. Immediately, 10 mL of ice-cold RPMI was added to terminate lysis and centrifuged (6 min, 1100 rpm), and the cell sediment was washed once more with isolation buffer to remove erythrocyte debris. Primary CD8+ T lymphocytes were positively isolated from spleens according to the instructions of the Dynabeads FlowComp Mouse CD8 Kit (Thermo Scientific, Waltham, MA, USA).

### 4.3. Cell Culture

Primary CD8+ T cells (1 × 10^6^/mL) were cultured in AIM V medium with 10% FCS, 50 μM BME, and 100 U/mL IL-2, then activated using anti-CD3e/CD28 beads (1:0.8 ratio). Cells were maintained in 24-well plates (37 °C, 5% CO_2_) (Bzeih et al., 2016) [41], passaged after 2 days with the addition of fresh complete medium, and used on days 3–5. For experiments, cells were either used immediately after bead removal or rested for 2 h before re-stimulation (triggering GzmB/IFNγ release). For FACS, days 3–5 CTLs were re-stimulated with plate-bound anti-CD3e (10 μg/mL) [29] for 0–24 h to assess IFNγ expression.

### 4.4. Immunocytochemistry

CTLs were stained for endogenous IFNγ using anti-IFNγ antibody. Cells were fixed in ice-cold 4% PFA for 10 min, washed with DPBS + 0.1 M glycine, permeabilized (0.1% Triton-X 100, 20 min), and blocked (5% BSA, 20–30 min). Staining was performed with rat anti-mouse IFNγ (1:200) and secondary anti-rat antibodies (1:1000) or IFNγ-Alexa488 (1:200), followed by SIM imaging. Antibodies are listed in Supplementary Materials Table S1.

### 4.5. Structured Illumination Microscopy

#### Structured Illumination Microscopy Setup

The SIM setup was from Zeiss (ELYRA PS.1). Images were acquired using a 63× Plan-Apochromat (NA1.4) objective with excitation light of 488, 561, and 647 nm and then processed for SIM to obtain higher resolutions. Z-stacks of 200 nm step size were used to scan cells. Zen 2012 software (Zen 2012; Carl Zeiss, Singapore) was used for the acquisition and processing of the images for higher resolutions.

### 4.6. Colocalization Analysis

For colocalization analysis, the JACoP plugin (Bolte 2006) [42] of Fiji (Johannes, 2012) [43] was used. Pearson’s and Manders’ overlap coefficients (Manders et al., 1993) [44] were used for quantification of the degree of colocalization.

### 4.7. Flow Cytometry

#### Intracellular IFNγ Measurement

WT CTLs were washed with DPBS (1×) after re-stimulation for 0.5 h, 1 h, 2 h, 3 h, 4 h, 6 h, 8 h, 12 h, and 24 h with plate-bound anti-CD3e antibody (10 μg/mL). The pellet (0.5 × 10^6^ CTLs) was resuspended in 250 μL fixation buffer and incubated on ice for 10 min using a BD Cytofix/Cytoperm™ Fixation/Permeabilization Kit (554714, BD Bioscience). Next, CTLs were centrifuged, resuspended in 100 μL wash buffer (1×), and stained for intracellular IFNγ and GzmB with rat anti-mouse IFNγ-Alexa488 (1:200) and rat anti-mouse GzmB-Alexa647 (1:200)) on ice (1 h). Cells were washed and measured by flow cytometry (FACS, BD FACSAria III). Data were analyzed using FlowJo v10.10.0_CL software (Celeza-Switzerland).

### 4.8. Surface Marker Analysis

CD8+ T lymphocytes, either with or without (re-)stimulation, were taken from cell culture and washed with cold DPBS (1×). Next, they were stained for CD62L, CD69, and CD25 using corresponding fluorescent antibodies without fixation (live staining, 30 min on ice). Next, cells were washed twice with chilled wash buffer (1×) and resuspended in cold DPBS (1×) for FACS analysis.

### 4.9. Degranulation Assay

Activated WT CTLs were washed and resuspended in AIM V after removing beads from the culture. Cells (0.2 × 10^6^/200 μL) were then cultured on a 10 µg/mL anti-CD3e-coated or DPBS (1×)-coated 96-well plate with 1 µL anti-CD107a-PE for 3 h at 37 °C for degranulation. The control cells were incubated without anti-CD107a-PE as a background signal. All cells were then washed twice with cold DPBS (1×) and checked by FACS. Data were analyzed using FlowJo software. Gates were set according to no-fluorescence control cells. Each independent experiment had a duplicate.

### 4.10. Statistical Analysis

For data analysis and to calculate the statistical significance, Fiji-win64, ImageJ-win64, Microsoft Excel (Microsoft), SigmaPlot 13, and GraphPad_Prism10.2.3 were used. The respective method to calculate statistical significance was given in the text for each figure. Figures were generated using FlowJo_v10.10.0_CL, GraphPad_Prism 10.2.3, Excel, and PowerPoint.

## 5. Conclusions

In conclusion, the expression of IFNγ and GzmB shows a maturation-dependent pattern but different kinetics. Unlike GzmB, IFNγ production is rapid and transient following TCR (re)activation and does not require mature immune synapse formation. We further demonstrate that CD62L downregulation and CD69 upregulation serve as robust markers of TCR-driven transition from central memory (T_CM_) to effector memory (T_EM_) phenotypes. Functionally, IFNγ may enhance CTL cytotoxicity by promoting Golgi-dependent processing and efficient sorting into lytic granules, with elevated IFNγ levels correlating with greater killing capacity. Additionally, we identify CRTAM as a critical early responder in primary CTL activation, where it may regulate initial IFNγ expression and contribute to IFNγ-mediated effector functions. Collectively, these insights establish a mechanistic framework for refining T cell-based immunotherapies against infectious diseases and malignancies.

## Data Availability

Additional data and information that support the findings of this study are available from the corresponding author upon reasonable request.

## Supplementary Materials

The following supporting information can be downloaded at: https://www.mdpi.com/article/10.3390/ijms26147024/s1.

## Abbreviations

CTL: CD8+ T lymphocyte; IFNγ: interferon-gamma; IFNγ^hi^: IFNγ^high^ subset; IFNγ^lo^: IFNγ^low^ subset; WT: wild-type; *GzmB-mTFP* KI: *granzyme B-mTFP* knock-in; TCR: T cell receptor; T_N_: naive CD8+ T cells; T_E_/T_EM_: effector/effector memory T cell; T_CM_: central memory T cell; T_EX_: T cell exhaustion; CGs: cytotoxic granules; SGs: secretory lysosomes; CD44: Homing Cell Adhesion Molecule; CD62L: L-selectin; CD25: Interleukin-2 receptor; CD127: IL-7 receptor alpha; LAMPs: lysosome-associated membrane glycoproteins; CD107a (LAMP-1), CD107b (LAMP-2), and CD63 (LAMP-3); FasL/CD95L: Fas ligand; Fas: Fas receptor; CRTAM: Class I-restricted T cell-associated molecule; DPBS: Dulbecco’s Phosphate-Buffered Saline; BME: β-Mercaptoethanol; IF: immunofluorescence; ICC: immunocytochemistry; FC: flow cytometry; TNF-α: tumor necrosis factor-alpha; IL-6: Interleukin-6; SIM: structured illumination microscopy; IRF-1: interferon regulatory factor-1; MFI: median fluorescence intensity; PCC: Pearson’s correlation coefficient.
