# Supplementary material for: IFNγ Expression Correlates with Enhanced Cytotoxicity in CD8+ T Cells
Source: Int J Mol Sci. 2025 Jul 21;26(14):7024. doi: 10.3390/ijms26147024 (PMC12295074; doi:10.3390/ijms26147024)
Supplement: Supplementary file 1 [file ijms-26-07024-s001.zip › ijms-3718460-supplementary.pdf]

Supplementary Data

Additional information

Extended Data Table S1 | Antibodies used in this study

Antibody, anti-	Supplier	Clone	Host	Dilution	Application
Mouse CD3e	BD Pharmingen	145-2C11	Hamster	1:100	Plate/glass coating
Mouse IFNγ	Biolegend	XMG1.2	Rat	1:200	FC, ICC
Mouse IFNγ-Alexa488	Biolegend	XMG1.2	Rat	1:200	FC, ICC
Mouse GzmB-Alexa647	Biolegend	GB11	Rat	1:200	FC, ICC
Mouse-CRTAM-PE	Biolegend	11-5	Rat	1:200	FC
Mouse-CD25-FITC	BD Pharmingen	7D4	Rat	1:200	FC
Mouse-CD44-PE	BD Bioscience	IM7	Rat	1:1200	FC
Mouse-CD62L-APC	BD Bioscience	MEL-14 (RUO)	Rat	1:1600	FC
Cis Golgi	BD Bioscience	GM 130	Mouse	1:100	ICC
Mouse-CD107a-PE	BD Bioscience	(1D4B)	Rat	1:200	FC
Mouse-CD69-APC	Thermo Scientific	H1.2F3	Hamst	1:100	FC
Mouse-IgG-Alexa647	Thermo Scientific	—	Goat	1:1000	ICC
Rat-IgG-Alexa647	Thermo Scientific	—	Chicken	1:800	ICC
Mouse-IgG-Alexa568	Thermo Scientific	—	Goat	1:400	ICC

Note: FC: flow cytometry, ICC: immunocytochemistry.

Extended Data

Extended Data Figure S1 | Dynamic expression profiles of CD44 and CD62L in reactivated CTLs. WT cytotoxic T lymphocytes (CTLs) activated for 3-5 days were re-stimulated with plate-bound anti-CD3e antibody (10 μg/mL) for the indicated durations (0, 0.5, 1, 2, 3, and 4 hours), followed by intracellular staining for IFNγ and granzyme B (GzmB). **(A, C)** Fold change in the percentage of CD44+ and CD62L+ cells. (**B,D)** Median fluorescence intensity (MFI) of CD44-PE (rat anti-mouse) and CD62L-APC (rat anti-mouse). Data represent mean ± SEM from ≥3 independent experiments. Statistical significance was determined by one-way ANOVA with post hoc unpaired t-tests: NS, $p > 0.05$; *$0.01 < p \leq 0.05$; **$0.001 < p \leq 0.01$; ***$0.0001 < p \leq 0.001$.

Extended Data Figure S2 | Flow cytometric analysis of T cell activation markers. WT cytotoxic T lymphocytes (CTLs) activated for 3–5 days were re-stimulated with plate-bound anti-CD3e antibody (10 μg/mL) for 0–4 hours and stained for surface expression of CD44 and CD62L. (**A)** Median fluorescence

intensity (MFI) of CD44-PE (rat anti-mouse) in CD44+ IFNγ^{hi} and IFNγ^{lo} CTLs. (**B**) MFI of CD62L-APC (rat anti-mouse) in CD62L+ IFNγ^{hi} and IFNγ^{lo} CTLs. Data represent mean ± SEM from ≥3 independent experiments. Statistical analysis was performed using one-way ANOVA followed by unpaired t-tests: NS, $p > 0.05$; *$0.01 < p \leq 0.05$; **$0.001 < p \leq 0.01$; ***$0.0001 < p \leq 0.001$.

Extended Data Figure S3 | Temporal dynamics of IFNγ and granzyme B expression in activated CTLs. WT cytotoxic T lymphocytes (CTLs) activated for 4 days were re-stimulated with plate-bound anti-CD3e monoclonal antibody (10 μg/mL) for 4–24 hours. Cells were subsequently stained for intracellular IFNγ and GzmB using fluorescently labeled antibodies and analyzed by flow cytometry (FlowJo v10.10.0). (**A**) IFNγ fluorescence intensity changes in IFNγ^{hi} and IFNγ^{lo} subsets. (**B**) GzmB fluorescence intensity changes in IFNγ^{hi} and IFNγ^{lo} subsets. Data represent mean ± SEM from 3 independent experiments. Statistical significance was determined by one-way ANOVA followed by unpaired t-tests: NS, $p > 0.05$, *$0.01 < p \leq 0.05$, **$0.001 < p \leq 0.01$, ***$0.0001 < p \leq 0.001$.

Extended Data Figure S4 | Temporal profiling of activation markers and IFNγ expression in primary CTLs. Day 0 WT cytotoxic T lymphocytes (CTLs) were stimulated in 96-well plates with plate-bound anti-CD3e (10 μg/mL) and anti-CD28 (5 μg/mL) antibodies for 0 h, 3 h, 6 h, 14 h, 24 h, 48 h,and 72 h , followed by surface staining for CRTAM, CD62L, CD69, and CD25, and intracellular staining for IFNγ. **(A)** Pseudocolor plots showing temporal expression patterns of CRTAM and IFNγ in day 0 CTLs following stimulation. **(D, G, J)** Pseudocolor plots depicting dynamic expression of CD62L, CD69, and CD25 in day 0 CTLs. **(B, E, H, K)** Quantification of CRTAM+, IFNγ+, CD62L+, CD69+, and CD25+ cell populations in stimulated day 0 CTLs. **(C, F, I, L)** Quantification of CRTAM-PE, IFNγ-Alexa488, CD62L-APC, CD69-APC, and CD25-PE median fluorescence intensity. Data represent mean ± SEM.

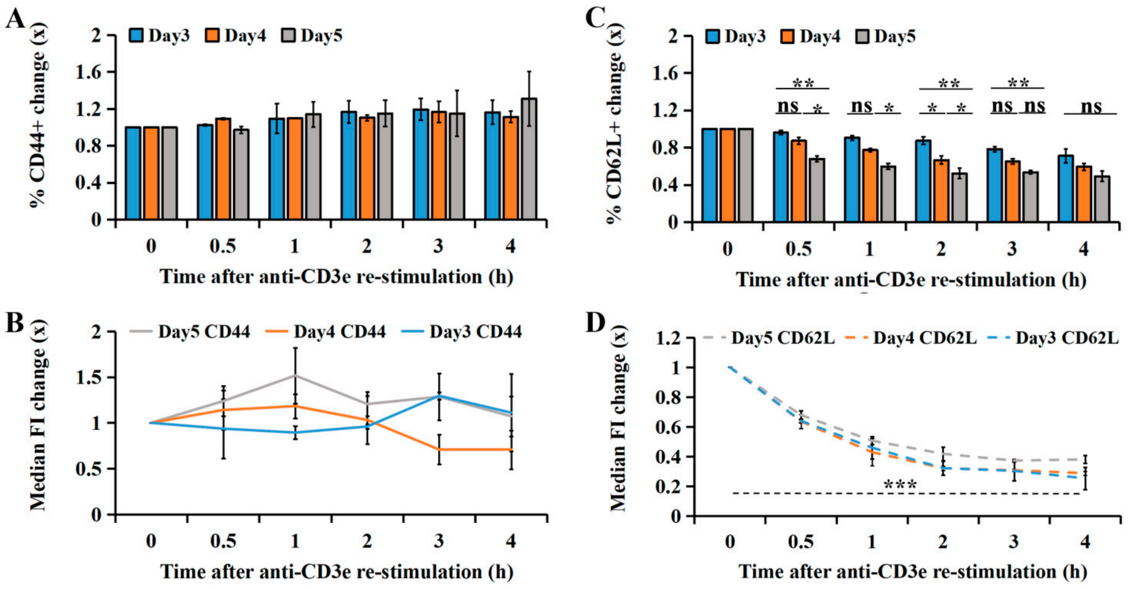

Extended Data Figure S1

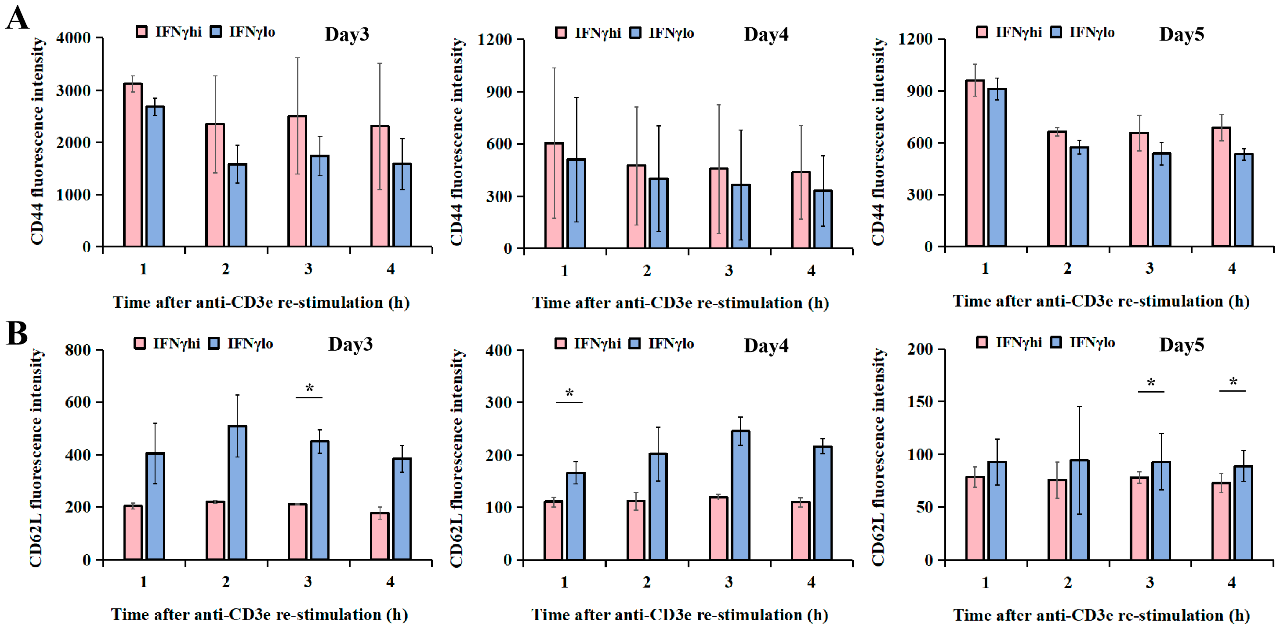

Extended Data Figure S2

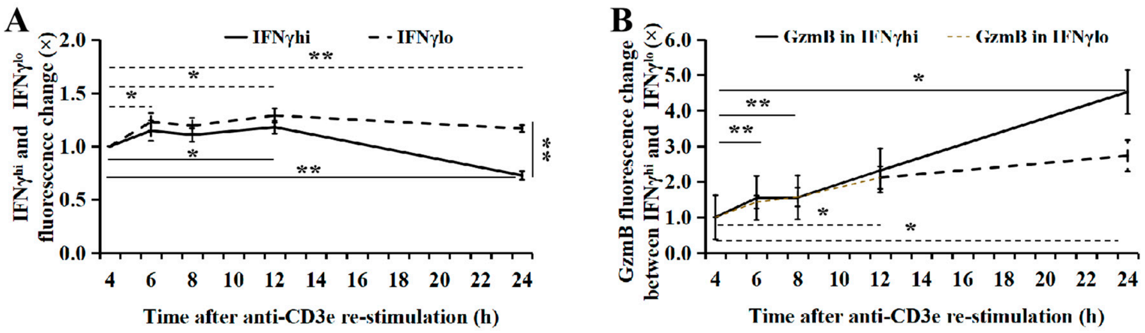

Extended Data Figure S3

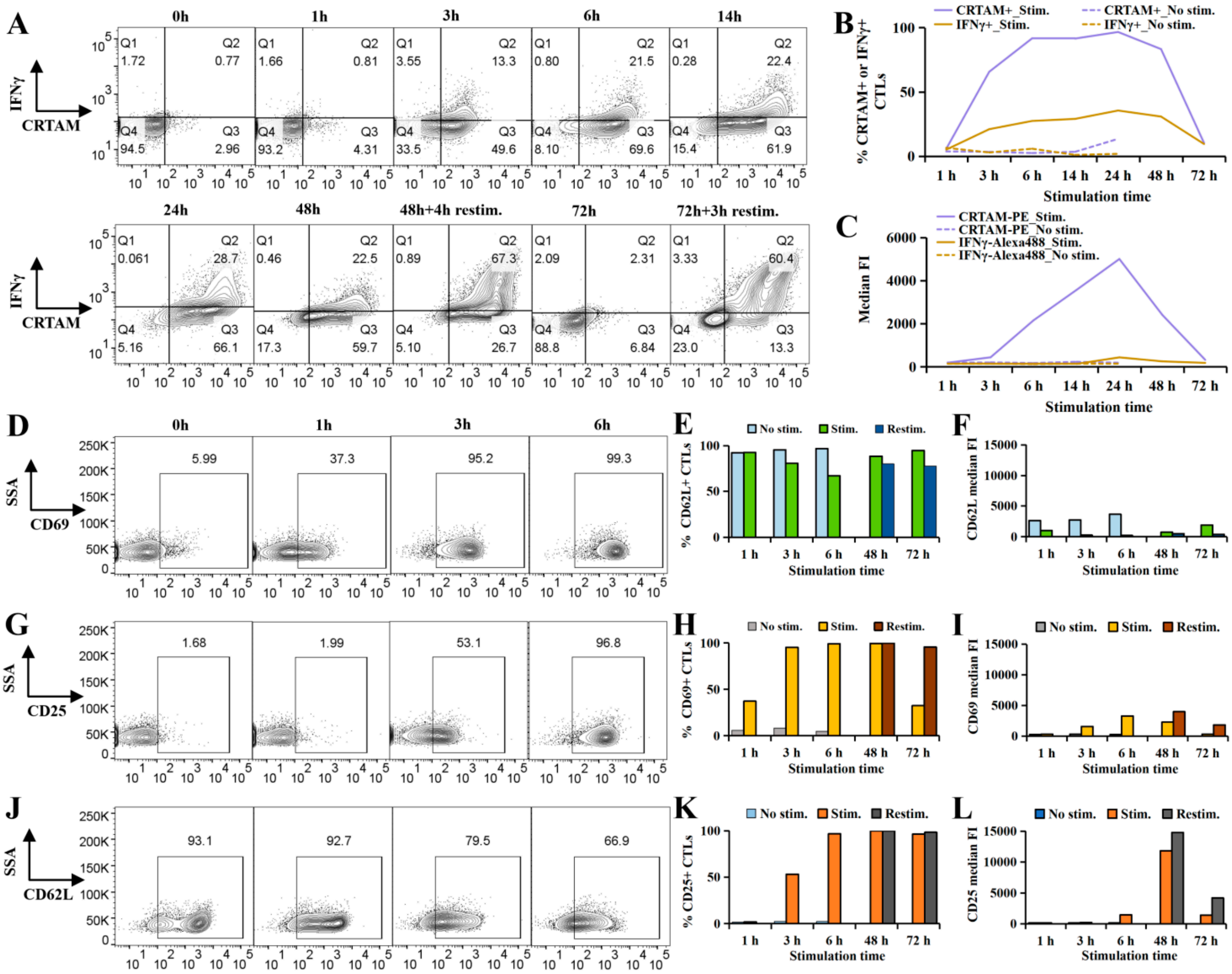

Extended Data Figure S4
